# Early increase in red cell distribution width-to-platelet ratio is associated with poor prognosis in sepsis patients: a retrospective cohort study

**DOI:** 10.3389/fmed.2026.1756060

**Published:** 2026-01-21

**Authors:** Dandan Gong, Keyang Li, Debao Li, Dongmei Ren, Zhenyao Wei, Nana Wu, Yuanyuan Wu, Junmin Li, Hongliang Dong

**Affiliations:** 1 Department of Clinical Pharmacy, Jiaozuo People’s Hospital, Jiaozuo, Henan, China; 2 Department of Clinical Laboratory, Jiaozuo People’s Hospital, Jiaozuo, Henan, China; 3 Department of Medical Oncology, Jiaozuo People’s Hospital, Jiaozuo, Henan, China; 4 Department of Obstetrics and Gynecology, Jiaozuo People’s Hospital, Jiaozuo, Henan, China

**Keywords:** 28-day in-hospital mortality, generalized additive mixed model (GAMM), longitudinal analysis, red cell distribution width-to-platelet ratio, sepsis

## Abstract

**Background:**

Previous studies have reported associations between baseline red cell distribution width-to-platelet ratio (RPR) and outcomes in sepsis patients. However, whether dynamic changes in RPR over time influence prognosis in sepsis remains unclear. This study aimed to investigate the significance of RPR trajectories during the progression of sepsis.

**Methods:**

This retrospective cohort study included sepsis patients admitted to the ICU from January 2014 to December 2015, using data extracted from the eICU Collaborative Research Database. Demographics, comorbidities, laboratory results, and clinical outcomes were collected. A generalized additive mixed model (GAMM) was employed to compare longitudinal trends in RPR between survivors and non-survivors, adjusting for potential confounders.

**Results:**

A total of 2,226 patients were included, with 328 deaths within 28 days of hospitalization. Scaled RPR exhibited divergent temporal patterns between survivors and non-survivors. In the first 6 days of ICU admission, scaled RPR gradually decreased and stabilized in survivors, whereas non-survivors showed a pronounced early increase. After adjusting for multiple covariates, this dynamic trend remained significant. The difference in scaled RPR between survivors and non-survivors widened at an average rate of 1.75 units per day.

**Conclusion:**

During the early stage of ICU admission (0–6 days), RPR may serve as a dynamic biomarker reflecting evolving pathophysiological changes. Early increases in RPR were observed to be associated with a higher risk of in-hospital mortality. These findings suggest that longitudinal RPR trajectories may provide supplementary information regarding risk stratification and warrant further investigation in conjunction with established clinical assessments.

## Introduction

Sepsis is a severe systemic inflammatory response to infection that progresses rapidly and frequently results in multiple organ dysfunction and even death (1, 2). It is not only one of the leading causes of mortality in critically ill patients but also imposes a substantial economic and social burden on healthcare systems worldwide (3, 4). Despite significant advances in the understanding of sepsis pathophysiology and improvements in therapeutic strategies, the incidence and mortality of sepsis remain alarmingly high, with an even greater impact in low- and middle-income countries (5). Therefore, early identification of high-risk patients is crucial to ensure timely and appropriate treatment.

Complex systemic pro-inflammatory and anti-inflammatory responses play key roles in the pathophysiological process of sepsis (6). Such immune dysregulation leads to multiple hematologic abnormalities, reflecting both the inflammatory burden and oxidative stress (7, 8). Red cell distribution width (RDW), an index reflecting variability in erythrocyte volume, is considered a sensitive marker of systemic inflammation and oxidative stress (9). Thrombocytopenia is also common in sepsis, although its underlying mechanisms are highly complex (10). Given that changes in RDW and platelet counts represent important components of hematologic pathophysiology during sepsis, their joint assessment may theoretically capture essential features of the disease process—namely, inflammation-driven erythrocyte heterogeneity and platelet consumption associated with inflammation and coagulation dysregulation.

Recently, a novel risk parameter combining RDW and platelet count—the RDW-to-platelet ratio (RPR)—has been applied to predict outcomes in patients with acute myocardial infarction, heart failure, ischemic stroke, and sepsis (11–14). Although several studies have examined the association between RPR and prognosis in sepsis patients (15, 16), data on the dynamic changes of this ratio over the disease course remain limited, despite their potential importance in assessing clinical deterioration. In this study, we aimed to investigate the temporal trends of RPR during the progression of sepsis.

## Subject and methods

### Data source

This study was reported in accordance with the Strengthening the Reporting of Observational Studies in Epidemiology (STROBE) guidelines (17). Data for this multicenter observational cohort were extracted from the eICU Collaborative Research Database (eICU-CRD), a publicly available, de-identified ICU database maintained by the Massachusetts Institute of Technology Laboratory for Computational Physiology. The eICU-CRD contains comprehensive clinical information from 208 hospitals across the United States and includes 200,859 ICU encounters from 139,367 unique patients between 2014 and 2015 (18). The database provides detailed records of vital signs, laboratory results, severity-of-illness scores, care plan documentation, admission diagnoses, and therapeutic interventions. Access to the eICU-CRD is regulated under the Health Insurance Portability and Accountability Act (HIPAA) Safe Harbor provision and has been certified as de-identified by Privacert (Cambridge, MA), ensuring strict protection of patient privacy and confidentiality. One of the authors, KL, completed the Collaborative Institutional Training Initiative (CITI) program for “Research with only data or specimens” (Certification ID: 40092459) and was granted the necessary authorization to access the database. Given the retrospective design of this study, the absence of any direct patient contact, and the exclusive use of de-identified data, the requirement for informed consent was waived. The study was conducted in accordance with the Declaration of Helsinki, and all methodologies complied with relevant ethical guidelines and regulatory standards.

### Study participants and data selection

Data extraction was conducted using PgAdmin PostgreSQL (version 17.4.1) and Navicat Premium (version 15.0.29). All patients diagnosed with sepsis at the time of ICU admission were eligible for inclusion. Sepsis was defined as suspected or documented infection plus an acute increase in Sequential Organ Failure Assessment (SOFA) score greater than 2 points (1) recorded in the Acute Physiology and Chronic Health Evaluation (APACHE) IV dataset (19). Infection was identified using ICD-9 codes available in the eICU Collaborative Research Database.

**Figure 1:**
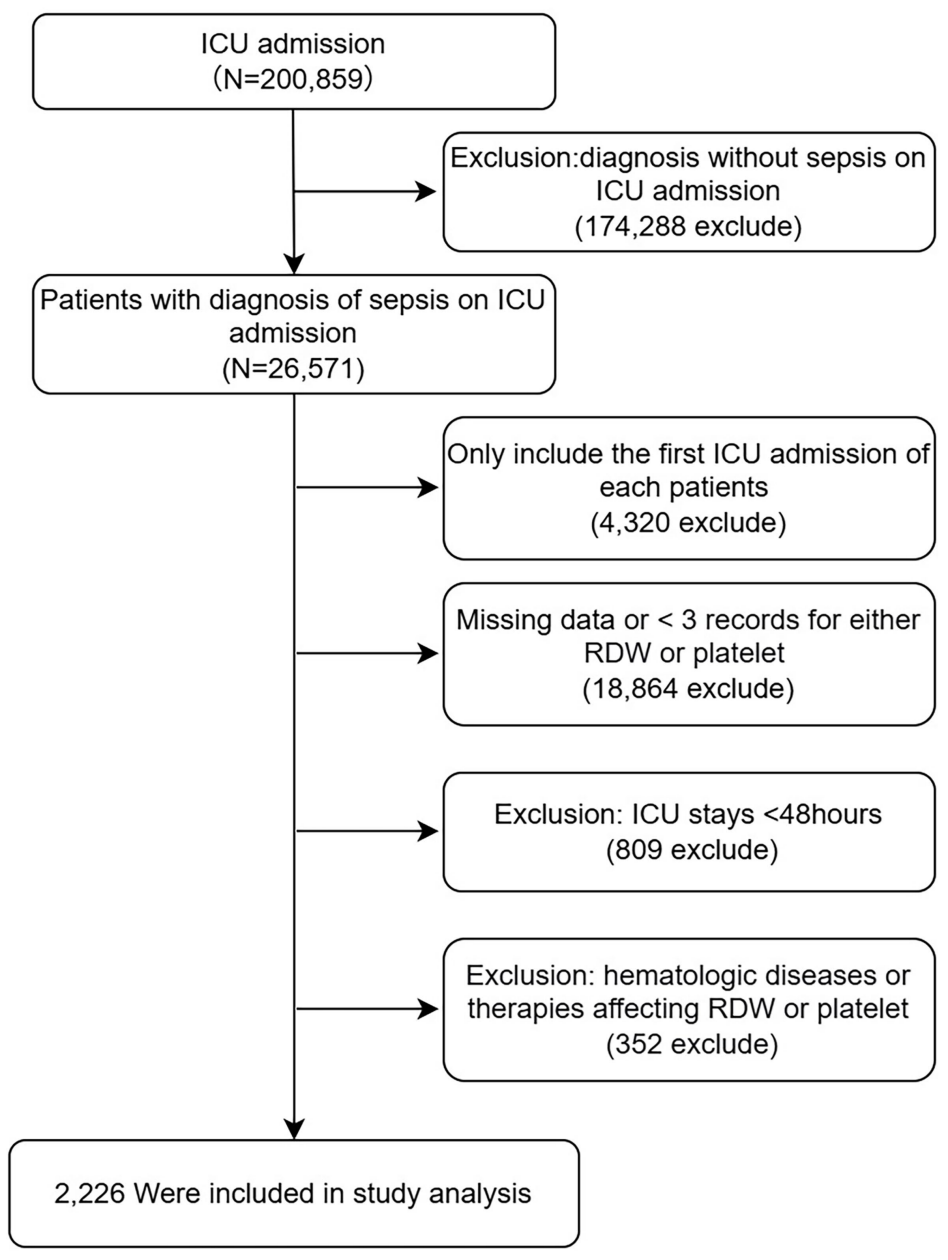
Flow chart for participants. ICU, intensive care unit; RDW, red cell distribution width.

Participants were excluded if they met any of the following criteria: (1) age <18 years; (2) fewer than three simultaneous measurements of RDW and platelet counts during the ICU stay; (3) receipt of treatments that may alter RDW or platelet levels, including thrombopoietic agents and erythropoiesis-stimulating agents (filgrastim, sargramostim, darbepoetin alfa, and epoetin alfa) or blood product transfusions (packed red blood cells and platelet concentrates) or the presence of underlying hematologic disorders that could affect RDW or platelet counts. For patients with multiple ICU admissions, only the first ICU stay was included in the analysis (Figure 1).

The extracted data included: (1) General clinical characteristics: age (in years), gender (male or female), race (categorized as White, others), BMI (kg/m^2^); (2) Laboratory values within 24 h of ICU admission: white blood cell (WBC, K/μL), platelet (PLT, K/μL), red cell distribution width (RDW,%), creatinine (mg/dL), BUN (mg/dL), glucose (mg/dL); (3) Comorbidities: chronic obstructive pulmonary disease (COPD), congestive heart failure (CHF), acute myocardial infarction (AMI), diabetes, pneumonia; (4) Treatments initiated within 24 h of ICU admission: use of mechanical ventilation, use of vasopressor medications, and dialysis; (5) Severity scores: Charlson comorbidity index (CCI), SOFA score at 24 h after ICU admission, Oxford Acute Severity of Illness Score (OASIS); (6) Outcome measures: hospital mortality within 28 days.

### Statistical analysis

**Figure 2:**
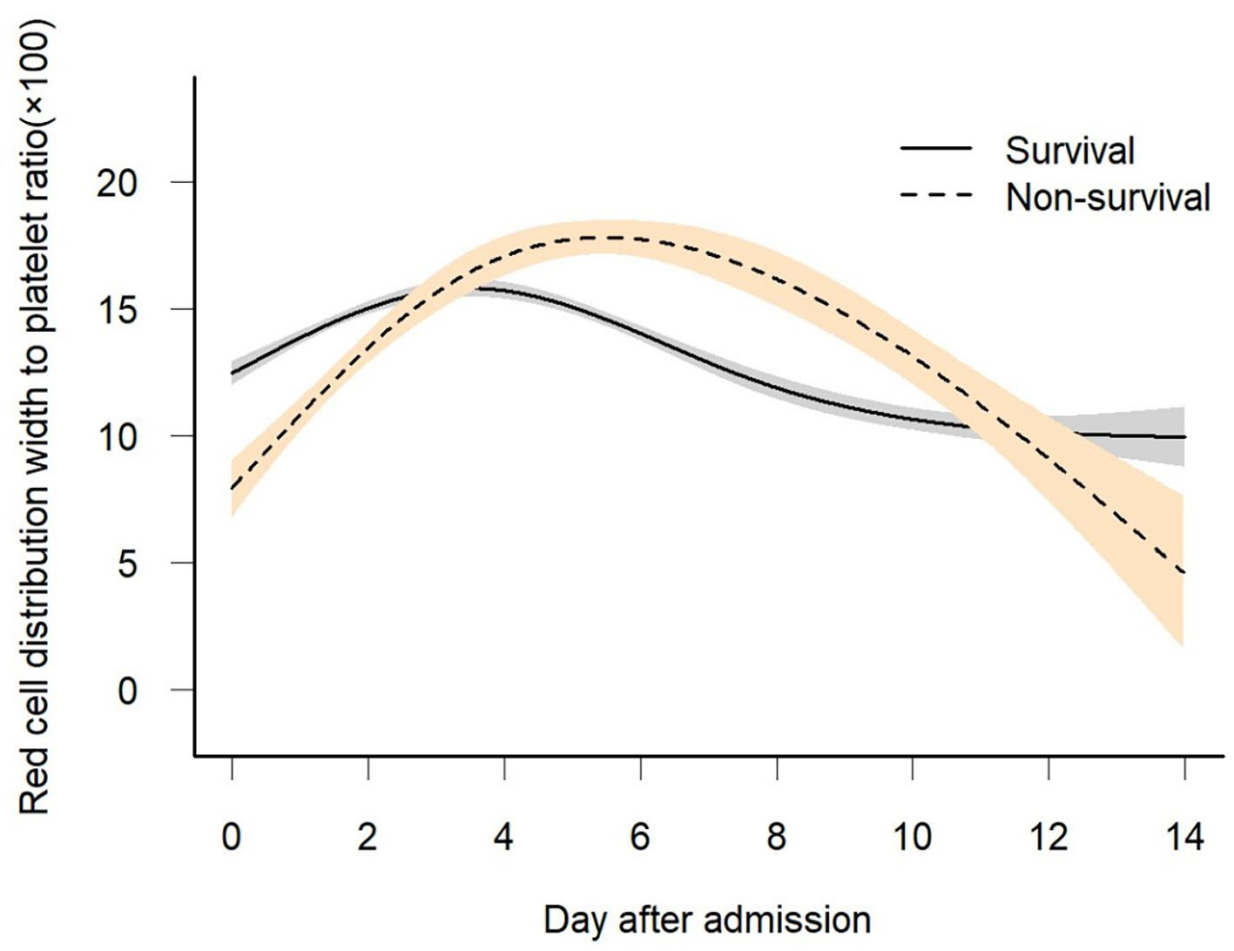
Association between dynamic change in scaled RPR over time and 28-day in-hospital mortality. A non-linear relationship was found between changes in scaled RPR over time and 28-day in-hospital mortality by GAMM. The smooth curve fitting graph shows the changes in scaled RPR of both survivors and non-survivors over time. The adjusted covariates include sex, age, BMI, baseline white blood cell, creatinine, BUN, glucose, mechanical ventilation, dialysis, vasopressor use, COPD, CHF, AMI, DM, pneumonia, Charlson comorbidity index, OASIS, APACHE score, and SOFA score.

Continuous variables were summarized as medians with interquartile ranges or as means with standard deviations (SD), as appropriate, while categorical variables were expressed as frequencies and percentages. The primary variable of interest in this study, the RDW-to-platelet ratio (RPR), was calculated as follows: RPR = RDW/platelet count. Because the resulting RPR values were numerically small, to improve interpretability, we analyzed scaled RPR values (raw RPR multiplied by 100). Baseline characteristics between survivors and non-survivors (Table 1) were compared using the Student’s *t*-test or the Kruskal–Wallis rank-sum test for continuous variables and the chi-square test for categorical variables. A generalized additive mixed model (GAMM) was employed to assess temporal changes in the RDW-to-platelet ratio between groups (Figure 2). Parallel GAMM analyses of red cell distribution width and platelet counts over the same period were also conducted, and the corresponding trajectories are presented in Supplementary Figures A,B. In addition, GAMMs were used to evaluate the association between early variation in RPR and mortality among patients with sepsis (Table 2). GAMMs have been demonstrated to be highly effective for analyzing repeated-measures data, particularly in the presence of missing observations, irregular measurement intervals, and moderate sample sizes (20, 21). In the GAMM analyses, time since ICU admission (DAY) was modeled as a continuous variable. Penalized cubic regression splines were applied to model nonlinear temporal trends when specified. Fixed-effect covariates included age, gender, mechanical ventilation, dialysis, vasopressor use, comorbidities (COPD, CHF, AMI, diabetes, and pneumonia), OASIS, APACHE score, SOFA score, and baseline laboratory variables (white blood cell count, creatinine, blood urea nitrogen, and glucose). To account for within-subject correlation arising from repeated measurements, a random intercept for each patient was included in all GAMM models. To contextualize the observed between-group differences relative to background biological and analytical variability, we conducted a variance-based sensitivity analysis. Within-subject standard deviations for RDW, platelet count, and RPR were calculated across repeated measurements for each patient. Standardized differences were then computed by dividing the between-group mean differences by the corresponding within-subject standard deviations, within-subject coefficients of variation were calculated to characterize effective temporal variability of RDW, platelet count, and RPR in ICU setting (22) (Supplementary Table 1). Models were fitted using maximum likelihood-based estimation as implemented. Missing longitudinal observations were not imputed; only complete observations for variables included in each model were analyzed. Model diagnostics included inspection of fitted values, residual distributions, and graphical evaluation of smooth terms. All statistical analyses were performed using R software (The R Foundation for Statistical Computing; https://www.r-project.org/) and EmpowerStats (X&Y Solutions, Inc., Boston, MA; https://www.empowerstats.com). Statistical significance was defined as a two-sided *p*-value <0.05.

**Table 1:** Baseline characteristics and clinical outcomes of patients.

Variable	Overall *N* = 2,226	Survivors *N* = 1898	Non-survivors *N* = 328	*p*-value
Gender (Male)	1,221 (54.9%)	1,041 (54.8%)	180 (54.9%)	1.000
Age (years)	63.5 ± 16.0	62.7 ± 16.1	68.0 ± 14.5	<0.001
Ethnicity				0.236
White	1,782 (80.1%)	1,511 (79.6%)	271 (82.6%)	
Others and unknown	444 (19.9%)	387 (20.4%)	57 (17.4%)	
BMI (kg/m^2^)	29.3 ± 9.8	29.5 ± 9.9	28.3 ± 8.9	0.038
WBC (K/μL)	13.8 (9.2–19.2)	13.6 (9.3–19.0)	14.6 (9.0–20.6)	0.416
Creatinine (mg/dL)	1.2 (0.8–1.9)	1.2 (0.8–1.8)	1.3 (0.9–2.0)	0.900
BUN (mg/dL)	26.0 (17.0–41.0)	25.0 (16.0–39.0)	33.0 (23.0–49.0)	<0.001
Glucose (mg/dL)	130.0 (103.0–171.0)	130.0 (103.0–170.0)	129.5 (101.5–175.0)	0.782
RDW (%)	16.1 ± 2.7	16.0 ± 2.7	16.7 ± 2.7	<0.001
Platelet (K/μL)	207.0 (143.0–292.0)	209.0 (144.0–294.0)	194.0 (129.0–274.0)	0.002
Scaled RPR	7.5 (5.3–11.1)	7.5 (5.3–10.8)	8.4 (5.9–13.1)	<0.001
SOFA score	7.0 (5.0–10.0)	7.0 (5.0–9.0)	9.0 (7.0–12.0)	<0.001
APACHE score	75.6 ± 26.5	73.0 ± 25.4	89.9 ± 28.5	<0.001
OASIS	33.7 ± 9.6	33.1 ± 9.5	37.1 ± 9.8	<0.001
Charlson comorbidity index	4.0 (2.0–5.0)	3.0 (2.0–5.0)	4.0 (3.0–6.0)	<0.001
Mechanical ventilation	938 (42.3%)	774 (41.0%)	164 (50.0%)	0.003
Dialysis	56 (2.5%)	54 (2.7%)	2 (0.5%)	0.027
Vasopressor use	48 (2.2%)	37 (2.0%)	11 (3.4%)	0.158
COPD	175 (7.9%)	146 (7.7%)	29 (8.8%)	0.547
Congestive heart failure	205 (9.2%)	170 (9.0%)	35 (10.7%)	0.735
Acute myocardial infarction	89 (4.0%)	64 (3.4%)	25 (7.6%)	<0.001
Diabetes mellitus	225 (10.1%)	214 (10.6%)	11 (5.4%)	0.055
Pneumonia	957 (43.0%)	809 (42.6%)	148 (45.1%)	0.433
ICU length of stay (days)	5.1 (3.2–9.1)	4.9 (3.1–8.8)	6.8 (4.2–10.6)	0.005
Hospital length of stay (days)	10.2 (6.8–15.9)	10.6 (7.0–16.5)	8.7 (5.5–13.3)	<0.001

Data are expressed as number (%) or mean ± SD or median (IQR).

WBC, white blood cell; RDW, red cell distribution width; SOFA, Sequential Organ Failure Assessment; OASIS, Oxford Acute Severity of Illness Score; COPD, chronic obstructive pulmonary disease, APACHE, Acute Physiology and Chronic Health Evaluation.

**Table 2:** Relationship between early (0–6 days) changes in RPR and in-hospital mortality (from GAMM).

Outcome	Model I		Model II	
	*β* (95% CI)	*p*-value	*β* (95% CI)	*p*-value
Intercept	14.87 (11.75, 18.00)	<0.0001	11.96 (7.64, 16.29)	<0.0001
Day	0.457 (0.301, 0.612)	<0.0001	0.459 (0.303, 0.614)	<0.0001
Death	5.56 (3.32, 7.81)	<0.0001	3.36 (1.19, 5.53)	0.0024
Day × Death	1.76 (1.36, 2.17)	<0.0001	1.75 (1.34, 2.15)	<0.0001

*β*, effect of RPR over time; CI, confidence interval; Intercept: RPR at day = 0 in the survival group (death = 0); Day: daily change in RPR in the survival group; Death: difference in RPR at day = 0 between non-survivors (death = 1) and survivors (death = 0); Day × Death: average daily difference in RPR change between non-survivors and survivors; Model I: adjusted for sex and age; Model II: adjusted for sex, age, BMI, baseline white blood cell, creatinine, bun, glucose, mechanical ventilation, dialysis, vasopressor use, COPD, CHF, AMI, DM, pneumonia, Charlson comorbidity index, OASIS, APACHE score, and SOFA score.

## Results

### Characteristics of participants

The demographic and clinical characteristics at baseline are presented in Table 1. Of the 2,226 included patients, 328 (14.7%) died during hospitalization. The proportion of males was nearly identical between survivors and non-survivors (54.8% vs. 54.9%, *p* = 1.000). Non-survivors were older than survivors (68.0 ± 14.5 vs. 62.7 ± 16.1 years, *p* < 0.001). The distribution of ethnicities was similar in both groups (*p* = 0.236). BMI was slightly lower in non-survivors (28.3 ± 8.9 vs. 29.5 ± 9.9 kg/m^2^, *p* = 0.038). Several laboratory parameters differed between groups. Although WBC and creatinine levels were comparable (*p* = 0.416 and *p* = 0.900, respectively), BUN values were markedly higher in non-survivors [33.0 (23.0–49.0) vs. 25.0 (16.0–39.0), *p* < 0.001]. Glucose levels showed no notable difference (*p* = 0.782). In contrast, non-survivors had higher RDW levels (16.7 ± 2.7 vs. 16.0 ± 2.7, *p* < 0.001) and lower platelet counts [194.0 (129.0–274.0) vs. 209.0 (144.0–294.0) K/μL, *p* = 0.002]. The scaled RPR was also elevated in patients who did not survive [8.4 (5.9–13.1) vs. 7.5 (5.3–10.8), *p* < 0.001].

Indicators of disease severity showed notable differences. Non-survivors had significantly higher SOFA scores [9.0 (7.0–12.0) vs. 7.0 (5.0–9.0), *p* < 0.001], APACHE scores (89.9 ± 28.5 vs. 73.0 ± 25.4, *p* < 0.001), and OASIS scores (37.1 ± 9.8 vs. 33.1 ± 9.5, *p* < 0.001). Similarly, the Charlson comorbidity index was higher among those who died [4.0 (3.0–6.0) vs. 3.0 (2.0–5.0), *p* < 0.001]. Non-survivors were more frequently treated with mechanical ventilation (50.0% vs. 41.0%, *p* = 0.003). Conversely, dialysis was less often used in these patients (0.5% vs. 2.7%, *p* = 0.027). The use of vasopressors did not differ significantly between groups (*p* = 0.158). Acute myocardial infarction was more common among non-survivors (7.6% vs. 3.4%, *p* < 0.001), while the frequencies of COPD, congestive heart failure, diabetes mellitus, and pneumonia were similar in both cohorts. Length of stay patterns also differed. ICU stays were longer among non-survivors [6.8 (4.2–10.6) vs. 4.9 (3.1–8.8) days, *p* = 0.005], whereas total hospital stays were shorter [8.7 (5.5–13.3) vs. 10.6 (7.0–16.5) days, *p* < 0.001].

In variance-based sensitivity analyses (Supplementary Table 1), the standardized differences between non-survivors and survivors were 2.19 for RDW, −1.63 for platelet count, and 6.15 for scaled RPR. These findings indicate that, at the population level, the observed group differences exceeded typical within-subject temporal variability.

### The association between temporal changes in the RDW-to-platelet ratio and in-hospital mortality was evaluated

Figure 2 shows the longitudinal trajectories of the RDW-to-platelet ratio (RPR) in survivors and non-survivors during the early phase (0–6 days) of ICU admission. Overall, RPR increased progressively over time in both groups; however, the rise was markedly steeper in non-survivors than in survivors. To further examine whether early (0–6 days) dynamic changes in RPR were associated with in-hospital mortality, we compared the temporal slopes between survivors and non-survivors. Table 2 demonstrates that the difference in RPR between the two groups widened significantly during the first 6 days of admission. In Model I, the interaction term indicated that non-survivors experienced an additional daily increase of 1.76 units in RPR compared with survivors. After adjusting for potential confounders—including sex, age, mechanical ventilation, dialysis, vasopressor use, COPD, congestive heart failure, acute myocardial infarction, diabetes mellitus, pneumonia, and SOFA score—the magnitude of this difference remained stable. In Model II, non-survivors still showed an excess increase of 1.77 units per day, confirming the robustness of the association.

## Discussion

In this study, we applied a generalized additive mixed model (GAMM) to investigate the temporal trajectory of the red cell distribution width-to-platelet ratio (RPR) during the clinical course of sepsis and its association with 28-day in-hospital mortality. Our findings revealed distinct longitudinal patterns between survivors and non-survivors. Specifically, scaled RPR declined over time and gradually stabilized in survivors, whereas non-survivors exhibited a marked early rise. Within the first six days after admission, the inter-group difference in scaled RPR reached 1.75 units, suggesting that suggesting that early divergence in this parameter may be indicative of different clinical outcomes.

Sepsis is defined as a systemic inflammatory response to infection, meeting at least two criteria of the systemic inflammatory response syndrome (SIRS) (23). Despite increasing understanding of the complex pro- and anti-inflammatory pathways involved, sepsis remains highly heterogeneous, and this heterogeneity has limited therapeutic progress. RDW has been associated with inflammation, oxidative stress, and impaired erythropoiesis. For example, in patients with chronic obstructive pulmonary disease (COPD), RDW correlates positively with C-reactive protein (*r* = 0.27, *p* < 0.001), supporting the notion that inflammation influences RDW fluctuations (24). Elevated RDW also reflects greater illness severity at ICU discharge and is an established predictor of adverse outcomes (25).

Platelets, traditionally recognized for their roles in hemostasis and thrombosis, are increasingly understood to participate actively in immunity, inflammation, and infection (26, 27). Their interaction with endothelial cells and circulating immune cells enables them to orchestrate multiple inflammatory processes (28). During sepsis, platelet activation contributes to endothelial injury, promotes neutrophil extracellular trap formation, and accelerates microthrombosis, thereby exacerbating sepsis-induced coagulopathy and inflammation. The resulting disseminated intravascular coagulation (DIC) can further drive organ dysfunction (29).

RDW undergoes dynamic changes across diverse clinical conditions, and recent evidence suggests that thrombocytopenia is associated with higher mortality than neutropenia, leukopenia, or leukocytosis (30). The RPR, a simple composite index derived from routine laboratory tests, integrates fluctuations in RDW and platelet count and has emerged as a novel marker of inflammation. Initially used to predict liver fibrosis in hepatitis patients (31), elevated baseline RPR has been associated with increased disease severity and poor prognosis in several disorders, including newly diagnosed glioblastoma (32), heart failure (12), and after coronary artery bypass grafting (33). However, most prior studies relied solely on baseline measurements. Given the rapidly evolving pathology of sepsis, static assessments may inadequately capture disease complexity. Recent research indicates that longitudinal biomarker trajectories may provide deeper insight into disease progression than single time-point measurements (34).

To address this gap, we incorporated time-dependent changes using a generalized additive mixed model (GAMM) to evaluate the association between RPR dynamics and 28-day mortality. After adjustment for potential confounders, non-survivors demonstrated a statistically higher daily increase in scaled RPR compared with survivors. Previous studies (35, 36) have reported that under controlled conditions, the short-term within-subject biological variation (CVI%) of platelet count is approximately 3.25 (2.81–3.85). For red cell distribution width (RDW), the short-term within-subject biological variation (CVI%) is approximately 0.37 (0.32–0.42). Notably, the magnitude of within-subject variability observed in this ICU cohort far exceeded biological CVI values reported under controlled conditions. This suggests that temporal fluctuations in RDW, platelet count, and RPR in critically ill patients reflect a composite of biological instability, treatment effects, disease progression, and analytical variability, rather than physiological day-to-day variation alone. In our variance-based analyses, population-level differences exceeded typical within-subject variability. From a clinical perspective, dynamic RPR trajectories should be interpreted as providing complementary, time-dependent information within an already high-risk population, rather than as an individual-level or standalone prognostic marker. These trajectories can complement established severity scores, such as SOFA or APACHE, by offering additional insights into a patient’s evolving condition. For instance, while SOFA and APACHE provide a snapshot of a patient’s status at a given point in time, dynamic RPR monitoring may capture subtle fluctuations that reflect more immediate changes in clinical condition, which may not be fully captured by static scores. However, it is important to emphasize that RPR trajectories reflect an association with mortality risk rather than a causal relationship. While dynamic RPR changes may help identify patients at higher risk, its independent prognostic value remains speculative and requires further validation in prospective studies. Thus, dynamic RPR monitoring should be seen as a complementary tool alongside existing severity scores, rather than a standalone predictive marker.

Nevertheless, several limitations should be acknowledged. First, the retrospective design of this study introduces the potential for inherent biases, missing data, and measurement variability in laboratory parameters. Second, despite multivariable adjustment, residual confounding cannot be entirely excluded, as non-survivors exhibited greater baseline illness severity and organ dysfunction, including higher blood urea nitrogen levels, a higher incidence of myocardial infarction, and an increased need for ventilatory support. These baseline clinical differences may partially contribute to the observed divergence in RPR trajectories and cannot be fully accounted for by statistical modeling alone. Third, important determinants of RDW and platelet counts were not captured in the database, including transfusion history beyond exclusion criteria, nutritional deficiencies (e.g., iron, folate, or vitamin B12 deficiency), occult bleeding, and bone marrow suppression, all of which may influence RPR dynamics and represent additional sources of residual confounding. Future prospective studies with more comprehensive clinical and laboratory data are needed to validate these findings. Finally, although the GAMM approach allows flexible modeling of nonlinear temporal patterns, it does not permit causal inference; therefore, RPR should be interpreted strictly as an associative rather than mechanistic parameter.

## Conclusion

In the early phase of sepsis, the red cell distribution width-to-platelet ratio (RPR) may reflect evolving pathophysiological changes. Our findings suggest that early increases in RPR are associated with higher risk of in-hospital mortality in sepsis patients. However, these observations should be regarded as hypothesis-generating, warranting further investigation into the role of RPR as a potential prognostic marker. Persistent or rapid increases in RPR may help identify patients at higher risk, but prospective studies are needed to confirm its clinical utility and applicability for guiding patient management.

## Data Availability

The raw data supporting the conclusions of this article will be made available by the authors, without undue reservation.
