# Supplementary material for: Early increase in red cell distribution width-to-platelet ratio is associated with poor prognosis in sepsis patients: a retrospective cohort study
Source: Front Med (Lausanne). 2026 Jan 21;13:1756060. doi: 10.3389/fmed.2026.1756060 (PMC12868120; doi:10.3389/fmed.2026.1756060)
Supplement: Supplementary file 1 [file Table_1.docx]

| **Marker** | **Mean difference (Non-survivors vs Survivors)** | **Median within-subject SD** | **Standardized difference** | **CV_I_ (%)**  **median** |
| --- | --- | --- | --- | --- |
| RDW | 0.85 | 0.39 | 2.19 | 2.46 |
| Platelet | -70.25 | 43.10 | −1.63 | 22.68 |
| Scaled RPR | 11.7 | 1.86 | 6.15 | 21.97 |

Supplementary Table1. Variance-based sensitivity analyses comparing between-group differences with within-subject variability and within-subject biological variation data

Note: Within-subject standard deviations were calculated across repeated measurements for each patient and summarized using the median. Standardized differences were calculated by dividing the between-group mean difference by the within-subject standard deviation. These analyses were conducted to contextualize observed group-level differences relative to background temporal variability. *CV_I_*_,_ within-subject biological variation.
