# Supplementary figures and images for: Early increase in red cell distribution width-to-platelet ratio is associated with poor prognosis in sepsis patients: a retrospective cohort study
Source: Front Med (Lausanne). 2026 Jan 21;13:1756060. doi: 10.3389/fmed.2026.1756060 (PMC12868120; doi:10.3389/fmed.2026.1756060)

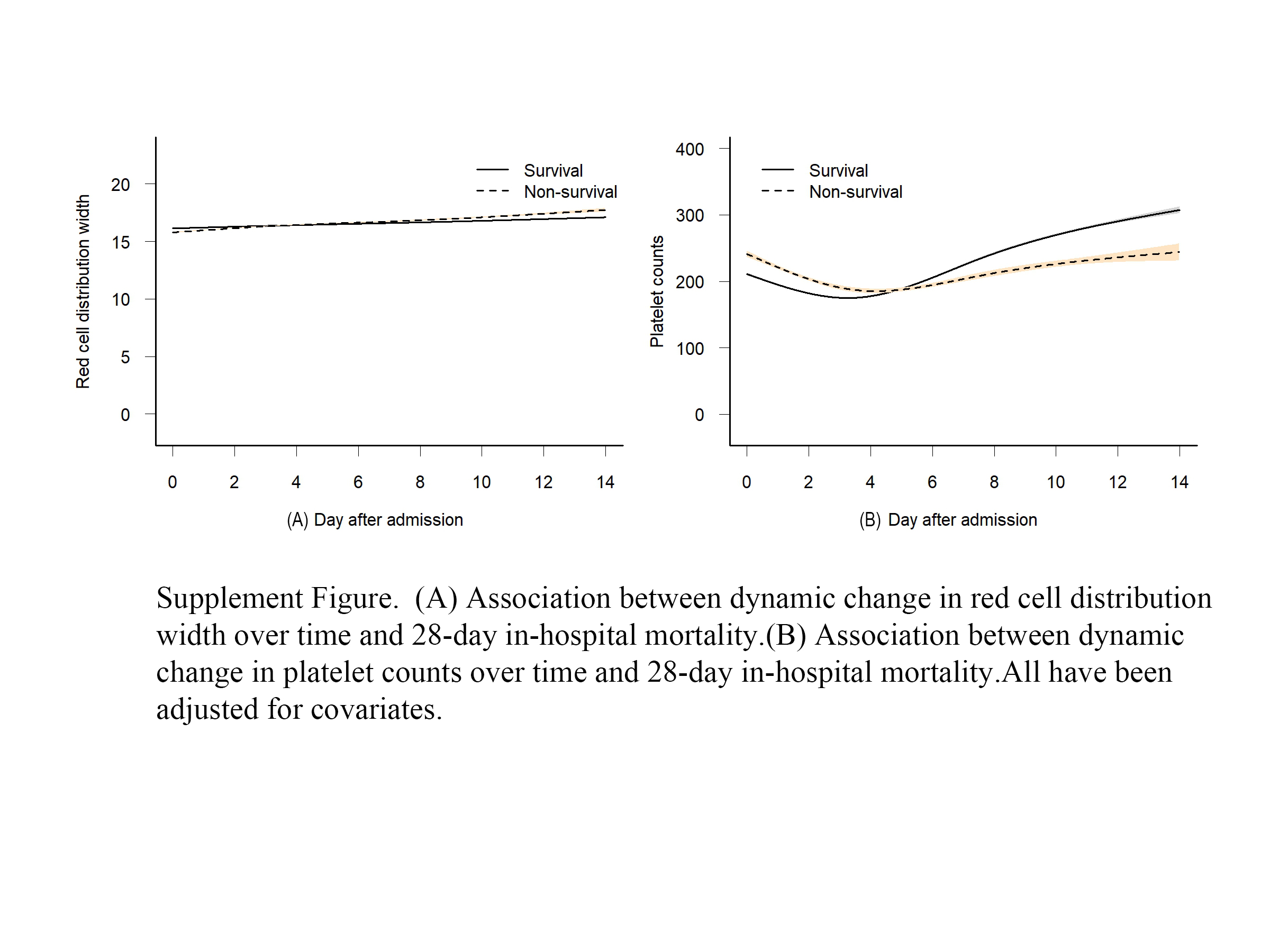

Supplement: Supplementary file 2 [file Image_1.jpeg]
